# Supplementary material for: Dynamic frame-by-frame motion correction for 18F-flurpiridaz PET-MPI using convolution neural network
Source: Eur J Nucl Med Mol Imaging. 2025 Nov 20;53(4):2708–20. doi: 10.1007/s00259-025-07660-x (PMC12834570; doi:10.1007/s00259-025-07660-x)
Supplement: Supplementary file 1 — Supplementary Material 1 (DOCX 3.39 MB) [file 259_2025_7660_MOESM1_ESM.docx]

**Dynamic frame-by-frame motion correction for ^18^F-flurpiridaz PET-MPI using convolution neural network**

Meghana Urs^1^, Aditya Killekar^1^, Valerie Builoff^1^, Mark Lemley^1^, Chih-Chun Wei^1^, Giselle Ramirez^1^, Paul Kavanagh^1^, Christopher Buckley^2^, and Piotr J. Slomka^1^

^1^Artificial Intelligence in Medicine Research Center, Departments of Biomedical Sciences, Medicine, and Cardiology, Cedars-Sinai Medical Center, Los Angeles, CA, United States

^2^GE Healthcare, Pharmaceutical Diagnostics, Buckinghamshire, England, UK

**Acknowledgements:** This research was supported in part by grant R35HL161195 from the National Heart, Lung, and Blood Institute/ National Institutes of Health (NHLBI/NIH) and R01EB034586 from the National Institute of Biomedical Imaging and Bioengineering (PI: Piotr Slomka). The content is solely the responsibility of the authors and does not necessarily represent the official views of the National Institutes of Health.

**First Author:**

Meghana Urs, MS

Cedars-Sinai Medical Center

6500 Wilshire Blvd, Los Angeles, California 90048

Phone: 424-315-0738

Email: [meghana.kiranurs@cshs.org](mailto:meghana.kiranurs@cshs.org )

First-author is not in training.

**Address for Correspondence:**

Piotr Slomka, PhD

Cedars-Sinai Medical Center

6500 Wilshire Blvd, Los Angeles, California 90048

Phone: 310-423-4348 Fax: 310-423-0173

Email: [Piotr.Slomka@cshs.org](mailto:Piotr.Slomka@cshs.org  )

ORCID iD: <https://orcid.org/0000-0002-6110-938X>

**Supplemental Material**

Study Population

The trial enrolled participants who could undergo either exercise or pharmacologic stress tests, who had undergone clinically indicated myocardial perfusion SPECT and invasive coronary angiography without any prior intervention and were of age 18 years and older(*1*). Exclusion criteria included individuals with a history of percutaneous coronary intervention, myocardial infarction, or other invasive coronary procedures within 6 months of ^18^F-flurpiridaz PET. Patients with current non-ischemic cardiomyopathy or a history of coronary artery bypass graft surgery were also excluded (*1*). Each study site obtained approval from their respective Institutional Review Board or ethics committee, and the study protocol adhered to the principles of the Declaration of Helsinki. Informed consent was obtained from all participants in writing prior to the commencement of any study procedures.

PET protocol

All imaging procedures followed the GE-265-001 PET Imaging Manual. PET imaging was performed using PET scanners (Supplemental Table 1) in 3D mode after participants fasted for a minimum of 3-hours. The protocol involved a rest/stress acquisition sequence. At rest, list-mode acquisition at a single bed position centered on the heart was initiated 10s before intravenous bolus injection of ^18^F-flurpiridaz (2.7 ± 0.2 mCi) followed by a 5–10 mL saline flush, and continued for 15 min. Pharmacologic stress was induced using regadenoson, adenosine, or dipyridamole, after which a second dynamic list-mode acquisition was performed beginning with a bolus injection of ^18^F-flurpiridaz (5.9 ± 0.3 mCi) and continued for 15 min. Both rest and stress acquisitions for a given subject were performed on the same scanner, with an average interval between doses of 53 ± 11 min.

List-mode data were reframed to generate a dynamic series (0–15 min) for time–activity curve (TAC) analysis and static images (5–15 min), with and without cardiac gating, for visual and semi-quantitative assessment. Raw data images were submitted to a central laboratory (BioClinica, Newtown, Pennsylvania) for quality control, reconstruction, and preparation for blinded read. Reconstruction included standard corrections (attenuation, scatter, randoms, deadtime and decay), according to designated time frames (15 × 10 s, 5 × 30 s, 5 × 60 s, 1 × 300 s). Protocol specifications for framing and reconstruction were standardized across all sites. No postfiltering was used.

All images were resampled to a common voxel spacing and normalized prior to model input to mitigate any residual scanner-specific variation. PET images underwent rigorous quality control before analysis.

Impact of Motion on MBF and MFR Corrections

Motion correction effects were strongly dependent on the magnitude of stress motion. ΔStress MBF increased progressively across motion tertiles, with the largest changes observed in patients with high stress motion (Supplemental Fig. 1a). In contrast, ΔRest MBF remained close to zero with only minor variability across tertiles. ΔMFR similarly showed increasing changes with higher stress motion, while rest motion was associated with much smaller shifts in MFR (Supplemental Fig. 1b). Spline analyses shown in Supplemental Fig. 2 confirmed these patterns: stress MBF corrections were minimal at low motion magnitudes but increased sharply when stress motion exceeded ~5 mm. ΔMFR followed a similar trajectory, rising steadily with greater stress motion. By comparison, rest MBF and MFR showed little systematic dependence on rest motion, with splines remaining nearly flat. Together, these findings demonstrate that stress motion is the dominant driver of bias in flow quantification, and that motion correction yields the greatest benefit in patients with substantial stress motion, while rest acquisitions are largely unaffected.

Standard clinical automatic motion correction

The standard automatic motion-correction (MC) algorithm corrected motion by aligning individual image frames to a static 3D geometrical model of the ventricles, defined by the left ventricle (LV) and right ventricle (RV) contours segmented from the sum of dynamic frames beyond the first 120 seconds using 3D rigid-body translations. Key frames were identified from the time-activity curves where well-defined correspondences between tracer distribution and regions in the geometric model were expected: an LV blood-pool peak frame, an LV blood-pool and myocardium crossover frame, and the end of acquisition frame where counts were concentrated in the LV myocardium only. The key frames were corrected by aligning them to the model through maximization of simple count-based similarity metrics developed for each. The in-between key frames were then corrected by registering them to synthetic reference frames generated from linear blending of neighboring key frames with variable ratios, which were derived from the time-activity curve through maximization of mutual information.

Deep learning-based automatic motion correction

Our network employed a 3D ResNet-based architecture, incorporating a regression head at the end to predict translation vectors. Each Conv3D block contained parametric rectified linear unit (PReLU) activation and batch normalization. Dropout (0.5) was also added to the fully connected layers to reduce overfitting. To train our network, different combinations of loss functions were explored. As the goal was to align each frame to the reference frame, mean square error (MSE) loss between predicted and target motion translation vectors was used (L_vec_). Manual operator motion corrections were performed on interpolated volumes which allow sub voxel translation. To accommodate the same in our training, the volumes were translated using trilinear interpolation. MSE loss was applied between volumes translated using predicted translation and target translation vectors (L_vol_). A combination of weighted (λ=0.5) L_vol_ and L_vec_ were used to update the network parameters.

Training dataset for AI motion correction

If an operator did not make any corrections, in which case the translation vector from that operator was (0,0,0), the translation vector from the other operator was used as the target. This ensures zero motion translations are chosen as targets only if both operators did not make any correction and that the model was exposed to realistic motion patterns and not dominated by zero-motion cases. To address potential discrepancies between operators, a third experienced operator reviewed the motion-corrected data and reached a consensus with each operator to reconcile the MC results. This quality control (QC) step ensured training consistency and minimized the influence of annotation outliers.

Since our data contained 21-26 dynamic frames and each patient had stress and rest scans, the total number of volumes were around ~9700-12,000. Data augmentation, which involved creating simulated motion vectors, was employed to drastically enlarge and diversify the dataset. Train and test splits were made on patients and not individual volumes ensuring all the dynamic frames belonging to one patient were in the same data split.

Data Preprocessing

PET scans acquired using different reconstruction parameters or from various scanners often exhibit varying appearances and voxel spacing. Consequently, it is essential to standardize and normalize the data before training or inference to ensure consistency and reliability in training and analysis. All volumes were resampled to same voxel spacing and cropped to a fixed size to achieve standardization. The cropped volumes were designed to include the region of interest by obtaining the bounding box around the LV mask while relatively incorporating the other three chambers and minimizing peripheral inclusion.

The AI model did not rely on myocardial segmentation for motion estimation. QPET-derived LV/RV contours were used only to define the approximate field of view, ensuring consistency with the manual correction workflow.

The cropped volumes were normalized using the z-score normalization ($z={x-\mu}/\sigma)$ method for scaling the intensity. Gaussian blur was applied to the volumes to calculate the mean(µ) and standard deviation(σ) used for z-score normalization.

Training and Testing Regimen

Each fold in the five-fold cross-validation process underwent specific data splitting procedures as follows: [1] a training split consisting of 147 or 148 patients used to train the model; [2] a validation split with 37 patients designated for tuning hyperparameters and ensuring no overfitting; and [3] a test split containing 47 or 46 patient scans used to evaluate the method. Results were derived by aggregating outcomes from all five test sets. Each test set included cases from sites that were not included in the network's training phase.

Adam optimizer (*2*) was employed with an initial learning rate of 10^-3^ and a weight decay of 10^-4^ to optimize network parameters. To prevent overfitting, ‘reduce on plateau’ scheduler was implemented, reducing the learning rate by a specific factor (10^-1^ in our case) if the validation loss failed to improve for 15 epochs (patience=15). Training continued until the learning rate reached 10^-6^ which was the minimum threshold in our experiment, and the model with the lowest validation loss was saved as the best model. Our architecture was implemented using Pytorch deep-learning framework. We trained our model on NVIDIA RTX 4090 GPU. The training approximately took 9 hours, batch size of 16 for a max of 400 epochs with early stopping and 5-fold cross validation.

Myocardial blood flow and Myocardial flow reserve quantification

The estimation of myocardial blood flow (MBF) in each coronary region relied on analyzing tracer uptake kinetics within the initial 90 seconds post-injection, assuming a first-pass extraction fraction of 0.94 (*3*). Rate-pressure product (RPP) was calculated as the product of heart rate and systolic blood pressure. Resting MBF values were then adjusted for RPP using the formula MBF_adj_ = MBF_rest_ / RPP_rest_ × 8500, where 8500 represents the RPP_ref_ value recommended by the Society of Nuclear Medicine and Molecular Imaging Cardiovascular Council and the American Society of Nuclear Cardiology (*4*). Stress and rest MBF values in mL/min/g were computed using parametric polar maps. Based on standard myocardial segmentation (*5*), seventeen segmental stress and rest MBF values were obtained from the polar map sample (*6*). Stress MBF at each segment was divided by the global rest MBF adjusted for RPP to calculate segmental myocardial flow reserve (MFR). Diagnostic evaluation on 231 patients indicated that minimal segmental stress MBF and MFR had superior diagnostic performance compared to global stress MBF and MFR (*7*). Therefore, minimal segmental stress MBF and MFR were used for diagnostic assessment. All MBF and MFR values were automatically derived in batch mode.

Statistical Analysis

In this study, the standard MC and DL-based MBF and MFR values were compared against the average of manual measurements from two independent expert operators to reduce random variability inherent to individual manual assessments. Additionally, we performed Bland–Altman analyses comparing standard MC and DL measurements against each reader individually (Supplemental Figs. 3 and 4). To further investigate performance across physiological conditions, we conducted separate analyses for rest and stress MBF. These analyses include individual concordance correlation coefficients (CCC) for both rest and stress MBF per operator (Supplemental Figs. 5 and 6). This separation avoids potential overestimation of agreement that may occur when rest and stress metrics—each with different dynamic ranges and variabilities—are combined. In addition, we report CCC values for MFR per operator to better characterize inter-reader agreement and model consistency across derived metrics (Supplemental Fig. 7).

**References**

**1.** Maddahi J, Lazewatsky J, Udelson J, et al. Phase-III Clinical Trial of Fluorine-18 Flurpiridaz Positron Emission Tomography for Evaluation of Coronary Artery Disease. *Journal of the American College of Cardiology.* 2020;76:391-401.

**2.** Kingma D, Ba J. Adam: A Method for Stochastic Optimization. *International Conference on Learning Representations.* 2014.

**3.** Huisman M, Higuchi T, Reder S, et al. Initial Characterization of an 18F-Labeled Myocardial Perfusion Tracer. *Journal of nuclear medicine : official publication, Society of Nuclear Medicine.* 2008;49:630-636.

**4.** Murthy VL, Bateman TM, Beanlands RS, et al. Clinical Quantification of Myocardial Blood Flow Using PET: Joint Position Paper of the SNMMI Cardiovascular Council and the ASNC. *Journal of Nuclear Medicine.* 2018;59:273.

**5.** Cerqueira MD, Weissman NJ, Dilsizian V, et al. Standardized myocardial segmentation and nomenclature for tomographic imaging of the heart. A statement for healthcare professionals from the Cardiac Imaging Committee of the Council on Clinical Cardiology of the American Heart Association. *Circulation.* 2002;105:539-542.

**6.** Slomka PJ, Alexanderson E, Jácome R, et al. Comparison of Clinical Tools for Measurements of Regional Stress and Rest Myocardial Blood Flow Assessed with ^13^N-Ammonia PET/CT. *Journal of Nuclear Medicine.* 2012;53:171.

**7.** Otaki Y, Van Kriekinge S, Wei C-C, et al. Improved myocardial blood flow estimation with residual activity correction and motion correction in 18F-flurpiridaz PET myocardial perfusion imaging. *European Journal of Nuclear Medicine and Molecular Imaging.* 2022;49.

**SUPPLEMENTAL FIGURES**


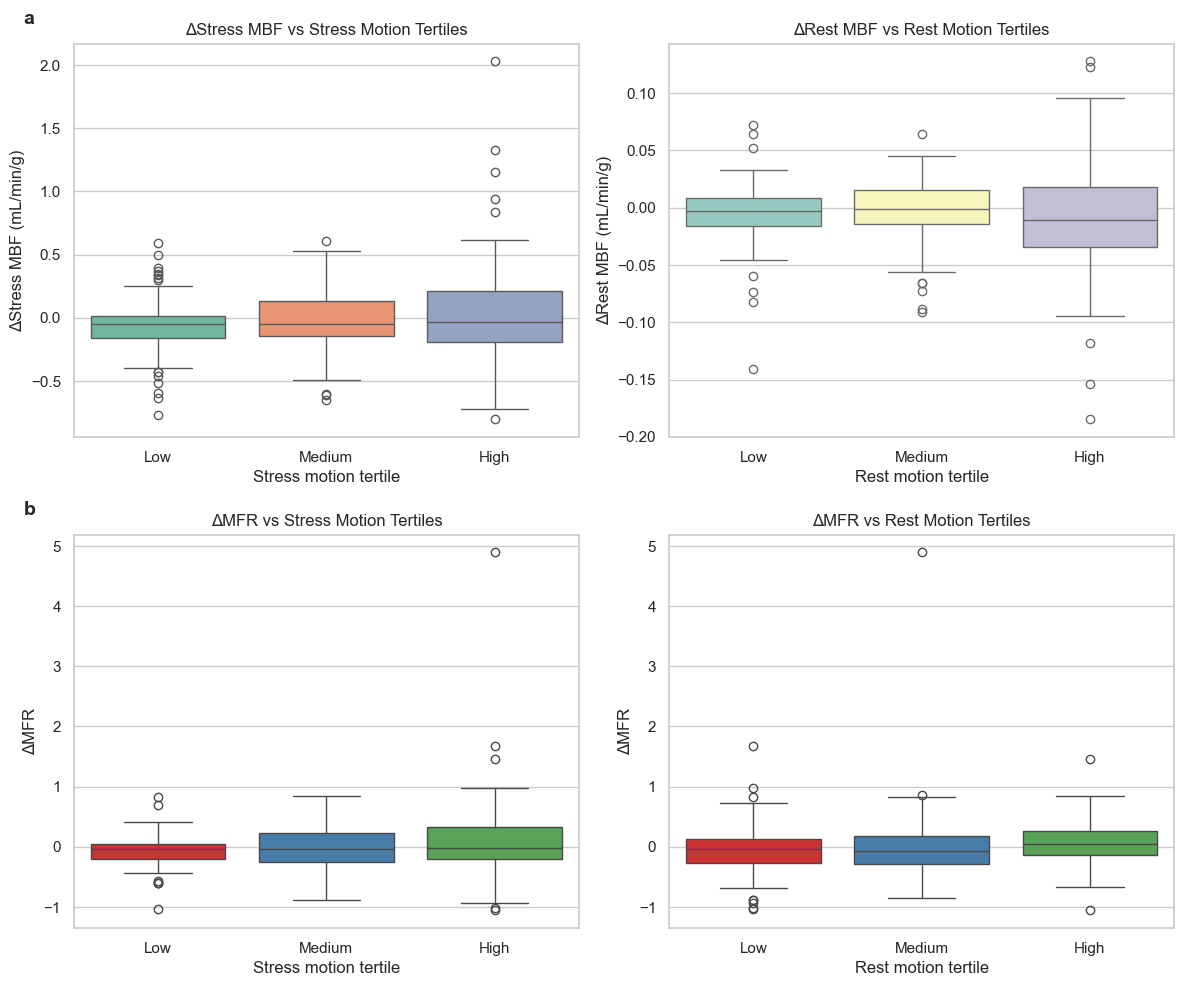
**SUPPLEMENTAL FIG. 1** Box plots depicting (a) ΔMBF vs motion tertiles (b) ΔMFR vs motion tertiles. ΔMBF: post - pre motion correction MBF, ΔMFR = post - pre motion correction MFR


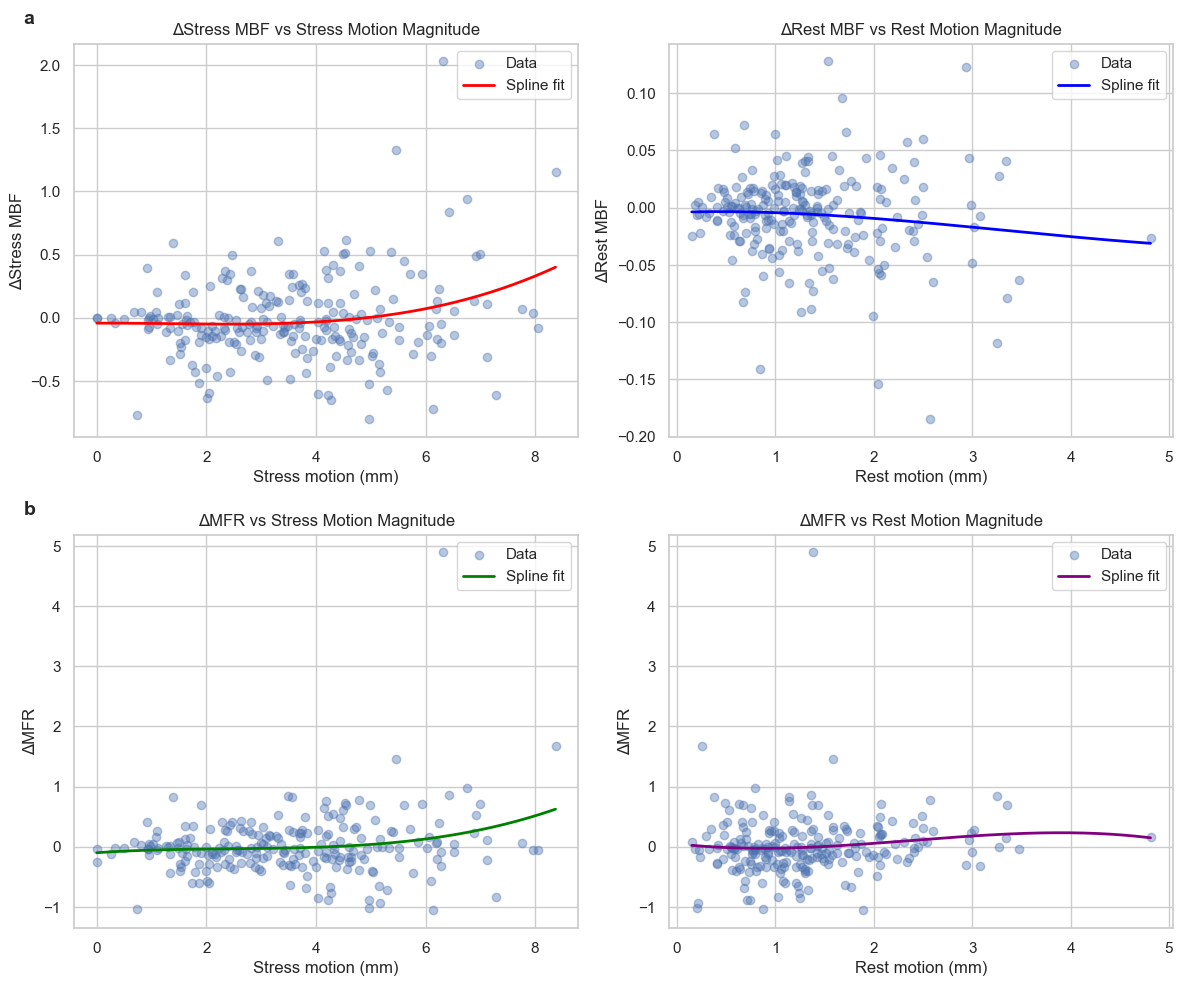
**SUPPLEMENTAL FIG. 2** Spline fit of motion magnitude vs (a) ΔMBF (b) ΔMFR. ΔMBF: post - pre motion correction MBF, ΔMFR = post - pre motion correction MFR


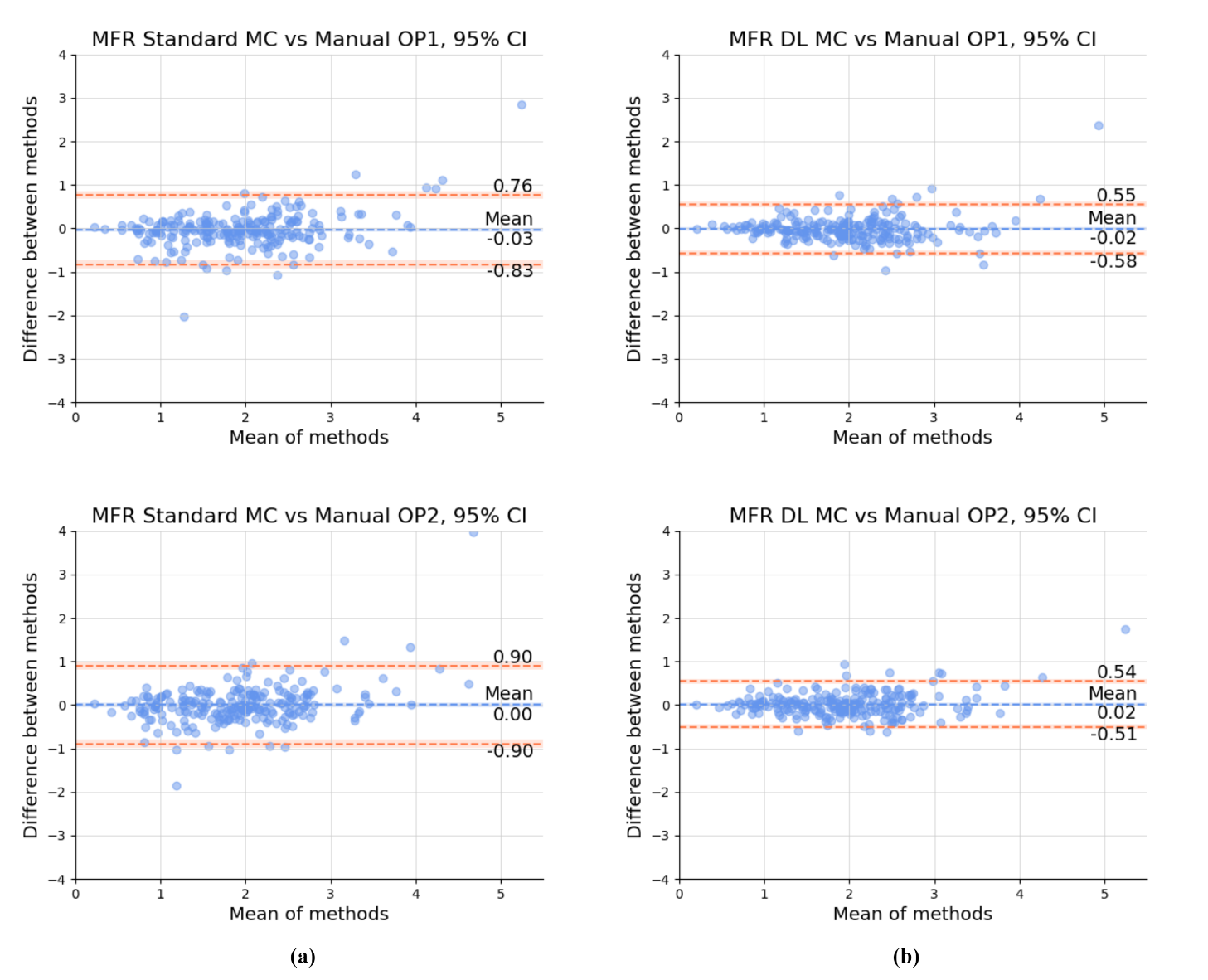
**SUPPLEMENTAL FIG. 3** Bland-Altman (BA) plots of (a) Standard non-AI automatic motion correction (MC) vs. individual operator manual MC and (b) Deep Learning (DL)-based MC vs. individual operator manual MC for myocardial flow reserve (MFR). The top row depicts BA plots for operator 1 (OP1) and the bottom row shows the BA plots for operator 2 (OP2). CI: Confidence interval


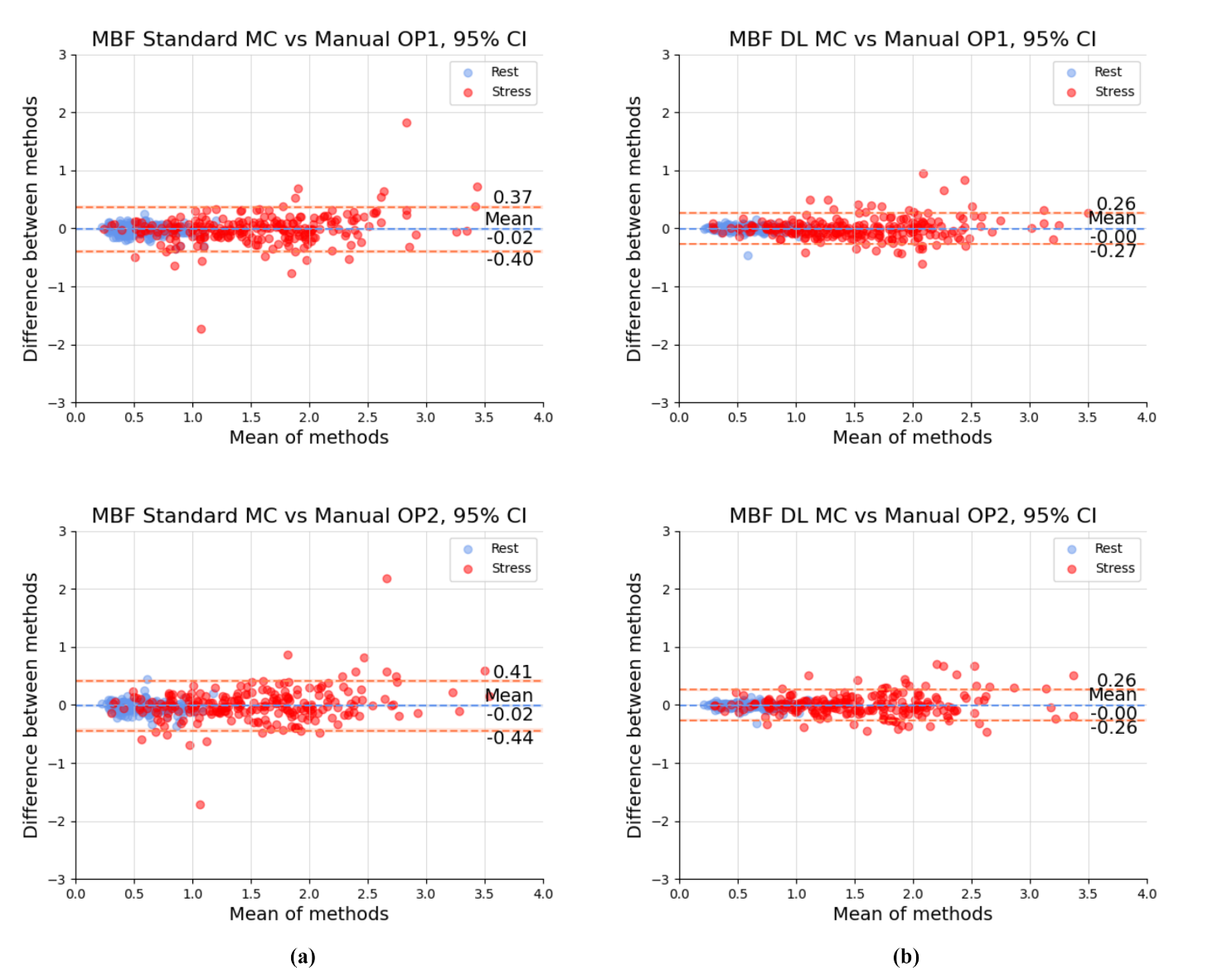
**SUPPLEMENTAL FIG. 4** Bland-Altman (BA) plots of (a) Standard non-AI automatic motion correction (MC) vs. individual operator manual MC and (b) Deep Learning (DL)-based MC vs. individual operator manual MC for stress and rest minimal segmental myocardial blood flow (MBF). The top row depicts BA plots for operator 1 (OP1) and the bottom row shows the BA plots for operator 2 (OP2). CI: Confidence interval


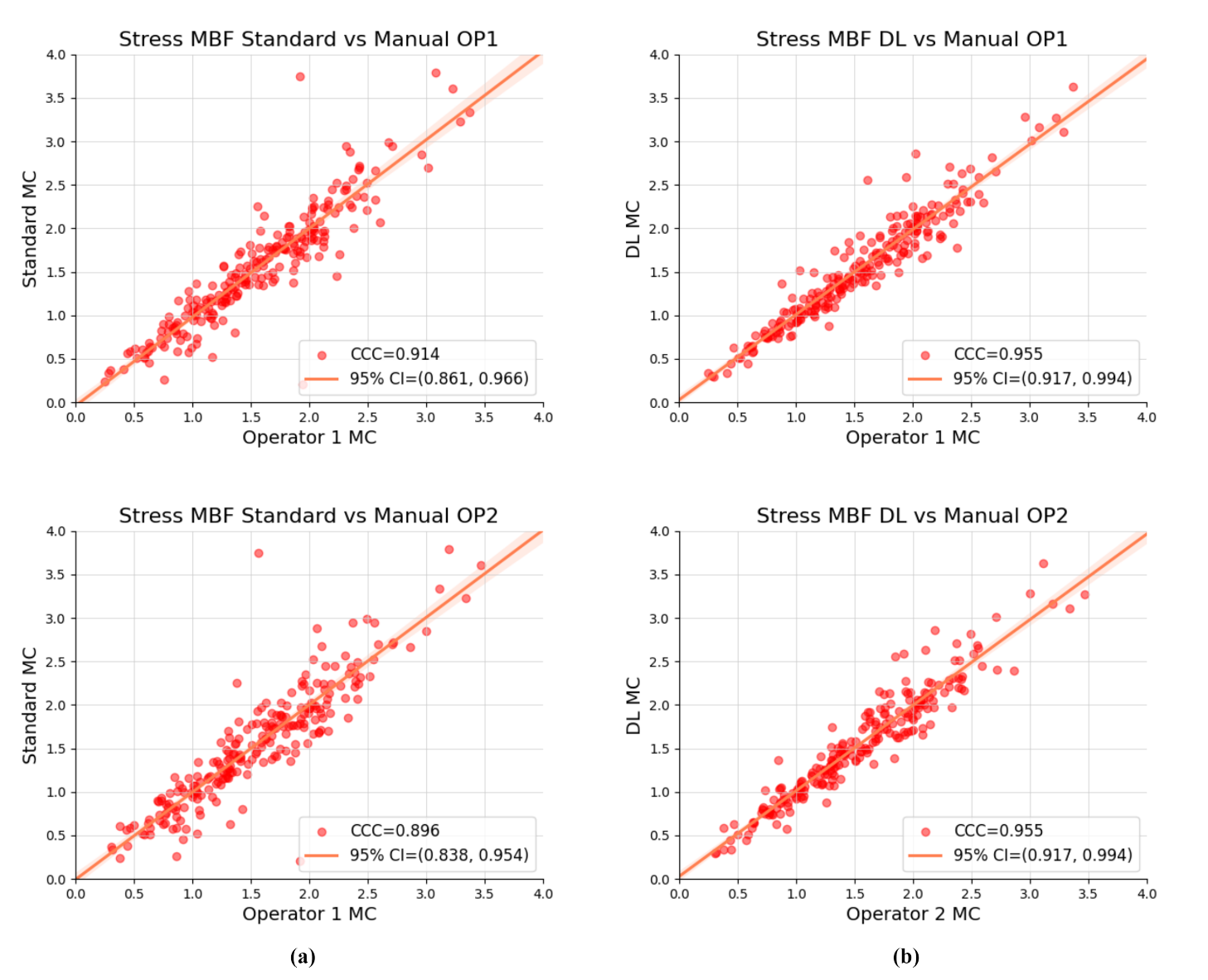
**SUPPLEMENTAL FIG. 5 Correlation plots of (a) Standard non-AI automatic motion correction (MC) vs. individual operator manual MC and (b) Deep Learning (DL)-based MC vs. individual operator manual MC for stress minimal segmental myocardial blood flow (MBF). The top row depicts Correlation plots for operator 1 (OP1) and the bottom row shows the Correlation plots for operator 2 (OP2). CI: Confidence interval**


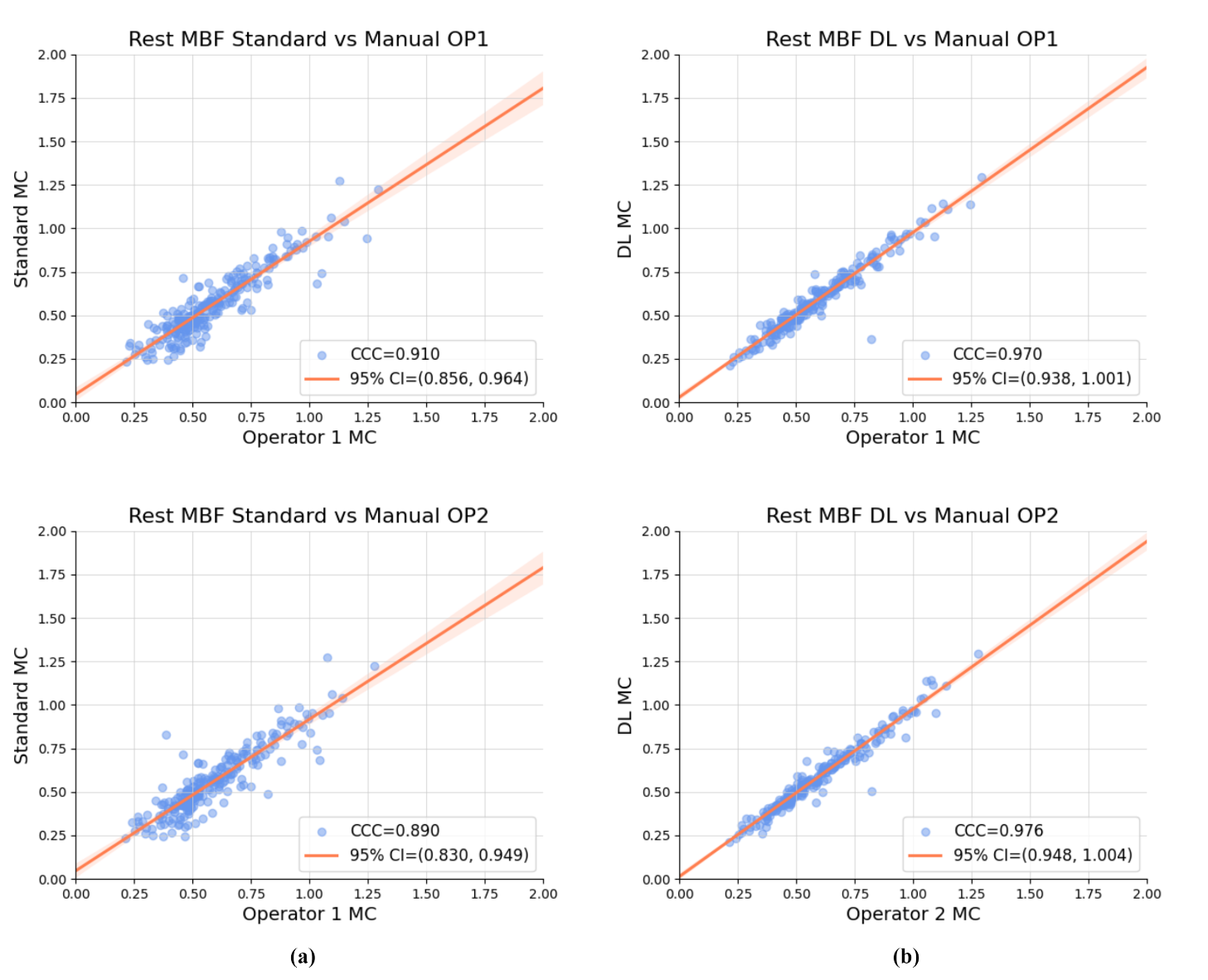
**SUPPLEMENTAL FIG. 6 Correlation plots of (a) Standard non-AI automatic motion correction (MC) vs. individual operator manual MC and (b) Deep Learning (DL)-based MC vs. individual operator manual MC for rest minimal segmental myocardial blood flow (MBF). The top row depicts Correlation plots for operator 1 (OP1) and the bottom row shows the Correlation plots for operator 2 (OP2). CI: Confidence interval**


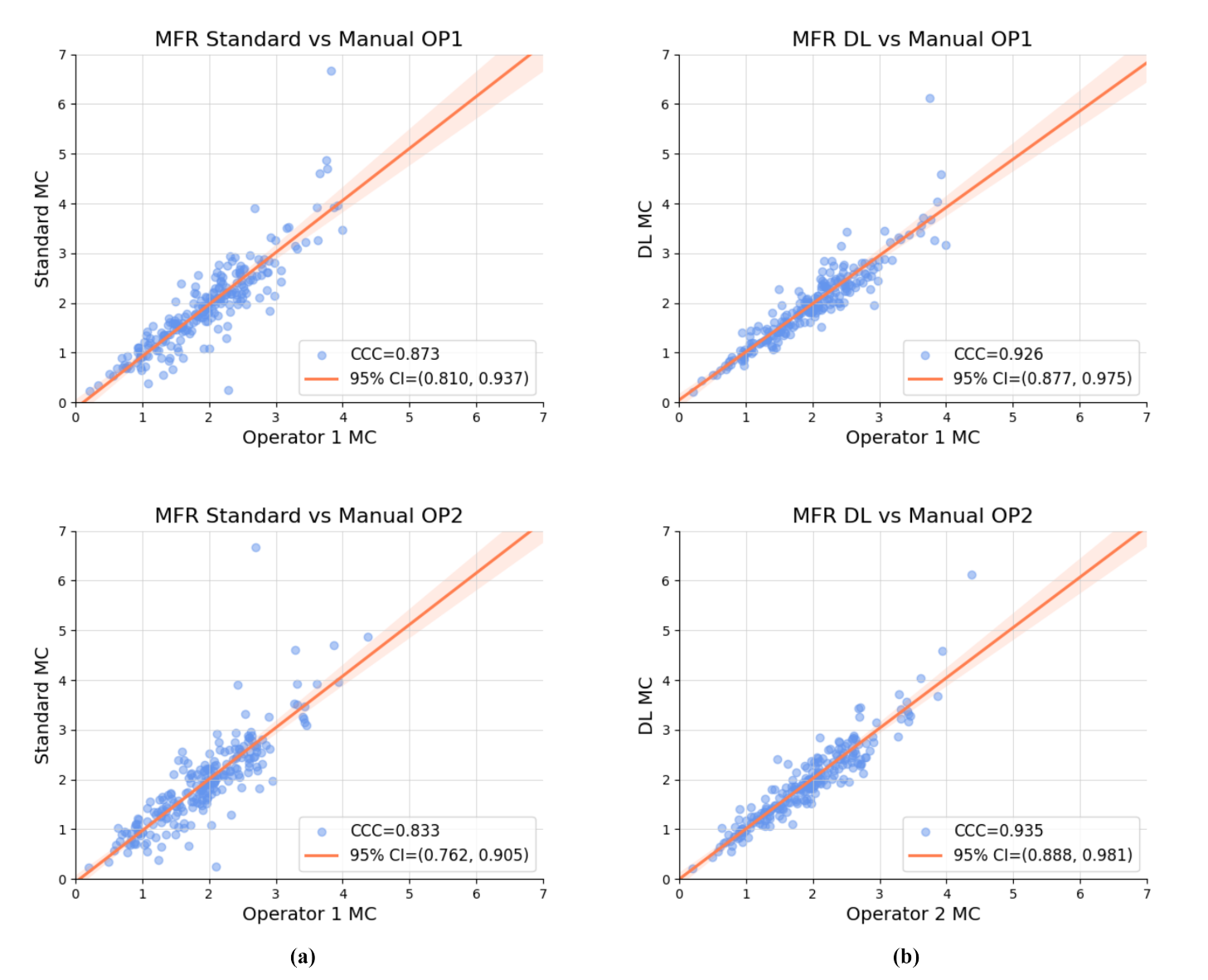
**SUPPLEMENTAL FIG. 7** Correlation plots of (a) Standard non-AI automatic motion correction (MC) vs. individual operator manual MC and (b) Deep Learning (DL)-based MC vs. individual operator manual MC for myocardial flow reserve (MFR). The top row depicts Correlation plots for operator 1 (OP1) and the bottom row shows the Correlation plots for operator 2 (OP2). CI: Confidence interval

**
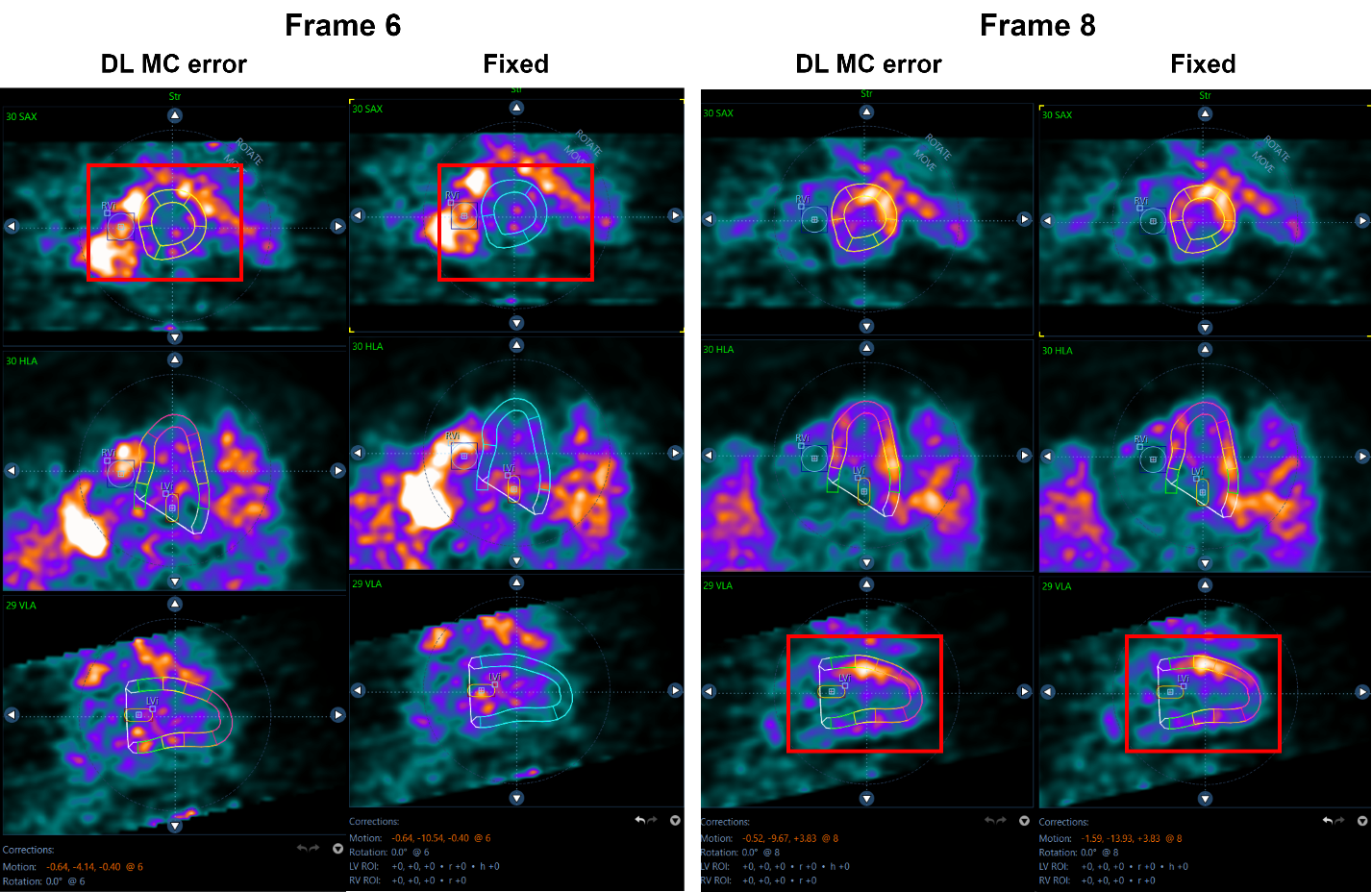
SUPPLEMENTAL FIG. 8** Example of an outlier in the Bland–Altman analysis of MFR. A small stress motion error in the early dynamic frames (red box) propagated into the MBF quantification. Because the rest MBF in this subject was low, the resulting error produced a disproportionately large relative deviation in MFR (>50% of the mean of the two manual measurements).


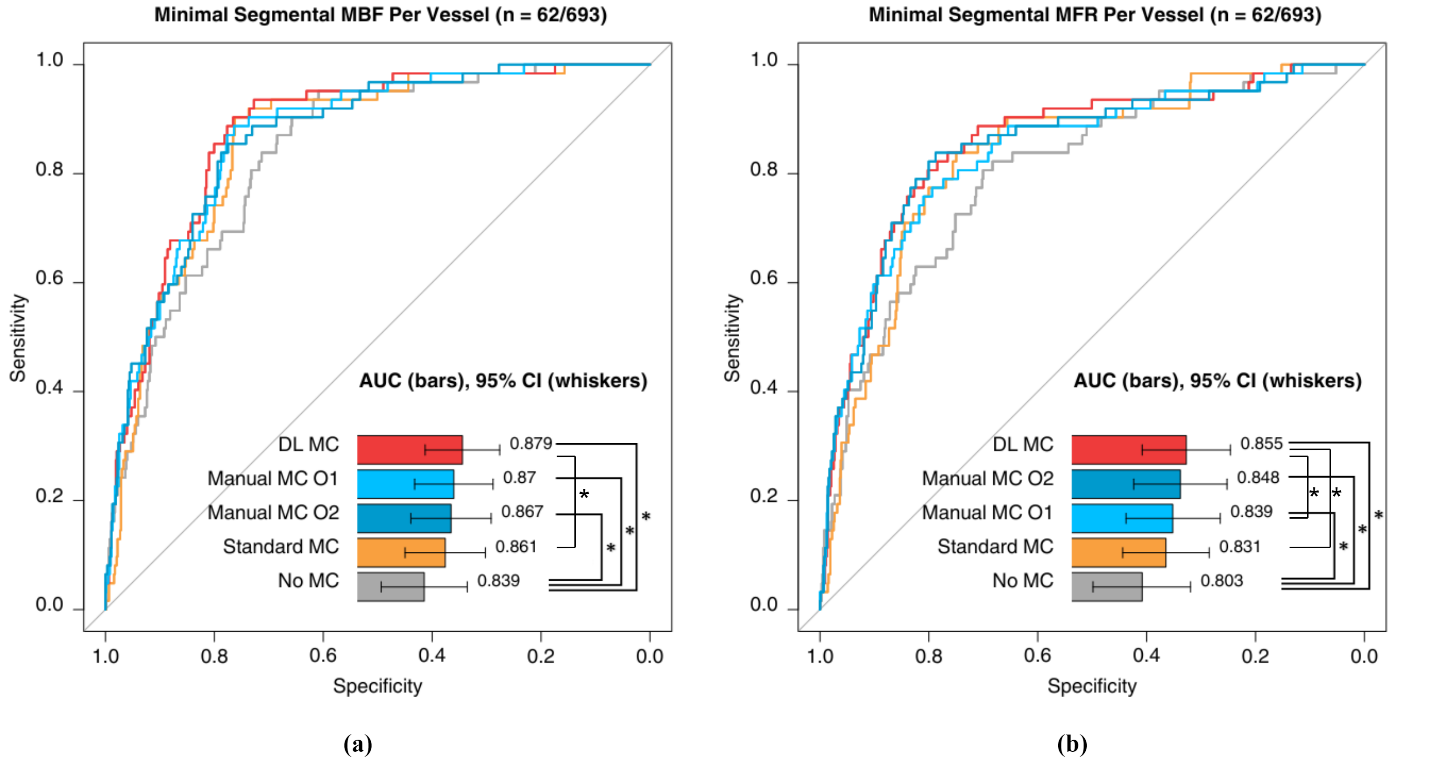
**SUPPLEMENTAL FIG. 9** Per-vessel diagnostic performance of minimal segmental (a) stress myocardial blood flow (MBF), (b) myocardial flow reserve (MFR) with residual activity correction applied, compared among manual motion correction (MC) from Operator 1 (O1) (light blue), Operator 2 (O2) (dark blue), standard non-AI automatic MC (orange), deep learning (DL) based automatic MC (red) and no-MC (grey). AUC: area under the receiver operating characteristic curve; CI: confidence interval; *: significant (p < 0.05)

**SUPPLEMENTAL TABLES**

SUPPLEMENTAL TABLE 1. PET camera models

| Model | Manufacturer | Number of cases |
| --- | --- | --- |
| Biograph TruePoint | Siemens | 87 |
| Discovery ST | GE Medical Systems | 46 |
| GEMINI TF TOF 64 | Philips Medical Systems | 36 |
| Discovery 690 | GE Medical Systems | 19 |
| Biograph40 | Siemens | 17 |
| BioGraph HiRes | Siemens | 8 |
| Biograph40_mCT | Siemens | 6 |
| Discovery 600 | GE Medical Systems | 4 |
| GEMINI TF TOF 16 | Philips Medical Systems | 3 |
| Biograph64 | SIEMENS | 2 |
| Discovery STE | GE Medical Systems | 2 |
| Advance | GE Medical Systems | 1 |
